# Aging-Associated Thyroid Dysfunction Contributes to Oxidative Stress and Worsened Functional Outcomes Following Traumatic Brain Injury

**DOI:** 10.3390/antiox12020217

**Published:** 2023-01-17

**Authors:** Cheng-Ta Hsieh, Ting-Lin Yen, Yu-Hao Chen, Jing-Shiun Jan, Ruei-Dun Teng, Chih-Hao Yang, Jui-Ming Sun

**Affiliations:** 1 Division of Neurosurgery, Department of Surgery, Sijhih Cathay General Hospital, New Taipei City 22174, Taiwan; 2 School of Medicine, National Tsing Hua University, Hsinchu 300044, Taiwan; 3 Department of Medicine, School of Medicine, Fu Jen Catholic University, New Taipei City 24205, Taiwan; 4 Department of Pharmacology, School of Medicine, College of Medicine, Taipei Medical University, No. 250, Wu Hsing St., Taipei 110, Taiwan; 5 Department of Medical Research, Cathay General Hospital, Taipei 22174, Taiwan; 6 Chung-Jen Junior College of Nursing, Health Sciences and Management, Chia-Yi City 62241, Taiwan; 7 Section of Neurosurgery, Department of Surgery, Ditmanson Medical Foundation, Chia-Yi Christian Hospital, Chia-Yi City 600, Taiwan; 8 Department of Biotechnology, Asia University, Taichung City 41354, Taiwan

**Keywords:** traumatic brain injury, aging, oxidative stress, melatonin, thyroid dysfunction

## Abstract

The incidence of traumatic brain injury (TBI) increases dramatically with advanced age and accumulating evidence indicates that age is one of the important predictors of an unfavorable prognosis after brain trauma. Unfortunately, thus far, evidence-based effective therapeutics for geriatric TBI is limited. By using middle-aged animals, we first confirm that there is an age-related change in TBI susceptibility manifested by increased inflammatory events, neuronal death and impaired functional outcomes in motor and cognitive behaviors. Since thyroid hormones function as endogenous regulators of oxidative stress, we postulate that age-related thyroid dysfunction could be a crucial pathology in the increased TBI severity. By surgically removing the thyroid glands, which recapitulates the age-related increase in TBI-susceptible phenotypes, we provide direct evidence showing that endogenous thyroid hormone-dependent compensatory regulation of antioxidant events modulates individual TBI susceptibility, which is abolished in aged or thyroidectomized individuals. The antioxidant capacity of melatonin is well-known, and we found acute melatonin treatment but not liothyronine (T3) supplementation improved the TBI-susceptible phenotypes of oxidative stress, excitotoxic neuronal loss and promotes functional recovery in the aged individuals with thyroid dysfunction. Our study suggests that monitoring thyroid function and acute administration of melatonin could be feasible therapeutics in the management of geriatric-TBI in clinic.

## 1. Introduction

Traumatic brain injury (TBI), as one of the leading causes of morbidity, disability and mortality, has been considered the most challenging public health problem worldwide. It has been estimated that over fifty million individuals suffer a TBI insult annually, and nearly 3.2 million TBI survivors suffer post-TBI complications, including neurological and psychological problems as well as long-term disability [1,2,3]. An increase in age is one of the important predictors of an unfavorable prognosis after brain trauma. Epidemiologically, elderly individuals over the age of 65 exhibit a higher incidence of TBI-related hospitalizations, emergency department visits, and readmission rates than the general population [4,5,6]. Statistically, geriatric TBI individuals at ages over 75 accounted for 26.5% of all TBI-related deaths and 31.4% of TBI-related hospitalizations in the United States [7,8]. Unsurprisingly, these geriatric TBI individuals displayed worse functional, cognitive, and psychosocial outcomes months or years post-brain injury [9,10]. Despite the growing epidemic knowledge of high incidence and severity of geriatric TBI, there are still limited evidence-based effective therapeutics for geriatric TBI [11,12]. There is an urgent need to understand its underlying pathological mechanisms and the development of feasible therapeutics for the growing geriatric TBI population.

Several pre-clinical rodent studies have highlighted the significance of aging in increased TBI severity [13,14,15,16,17]. Although numerous pathological abnormities have been correlated to the enhanced TBI-susceptible phenotypes in aged brains, confounding factors such as pre-existing biological function deficits and the impact of complications in the aged individuals might greatly interfere with the study of the underlying pathology and limit the development of feasible therapeutics. As the brain ages, the well-known underlying biological changes with increased cellular senescence [18], accumulation of DNA damage [19,20], dysregulated immune responses [21] and increased oxidative stress [22] could act as confounding factors that make it very difficult to isolate and interpret the underlying pathology of a geriatric TBI. To reduce the interference of the pre-existing factors from the aged brain, in the current study, we decided to use middle-aged animals between the ages of 9–12 months old, which display no obvious deficits in motor or cognitive performance and have similar levels in their basal inflammatory responses and oxidative stress to answer the pathology of TBI-susceptibility with increasing age.

The function of our endocrine system, comprising various hormonal-releasing organs, undergoes age-related changes that negatively impact its proper performance. The thyroid gland, which plays a vital role in the human body’s metabolism, growth and development also displays structural and functional alterations with aging [23,24,25]. Thyroid dysfunction (especially subclinical thyroid dysfunction) is common in the elderly. Accumulating evidence has indicated the prevalence of subclinical hypothyroidism rises with aging and ranges from 3 to 16% in the elderly at the age of 60 and older [26,27]. Interestingly, thyroid hormone has been reported to play important roles in oxidative regulation. Age-related thyroid dysfunction has been associated with oxidative stress in the pathology of numerous human diseases, such as obesity and neurodegenerative disorders [28,29,30,31]. In addition, excessive oxidative stress overload, which is the imbalance in pro-oxidants and antioxidants and consequently unwarranted production of reactive oxygen species, could result in profound neuronal damage and dampen functional recovery after brain trauma [32,33]. As thyroid dysfunction increases with age and its potential link to oxidative regulation, we hypothesize that the changes in thyroid hormones levels in the elderly might modulate TBI-related oxidative stress and functional outcomes.

Melatonin, the hormone secreted from the pineal gland, is well known to demonstrate a remarkable antioxidant capacity [34]. In addition, the amount and rate of its secretion decline gradually along with the increase in age that pathologically links to the progression of certain neurodegenerative diseases [35]. The robust antioxidant capacity of melatonin has been linked to its ability to scavenge oxygen free radicals and also the stimulation in the synthesis of anti-oxidative enzymes such as superoxide dismutase (SOD) and glutathione peroxidase (GPX). Based on these known properties and pathological association in aging-related neurodegeneration of melatonin, it is interesting to ask if the acute melatonin treatment might provide a neuroprotective impact on the TBI-susceptible phenotypes in the middle-aged individuals.

In the current study, by using the middle-aged animals, we aim to explore the pathology of aging-associated thyroid dysfunction in modulating oxidative stress, neurodegeneration and functional outcomes in geriatric-TBI and eventually develop feasible therapeutics for the treatment of TBI in aged individuals with thyroid dysfunction.

## 2. Materials and Methods

### 2.1. Animals

Male C57Bl/6 mice (adult: 9–12 weeks old, middle-aged: 36–40 weeks old) were purchased from BioLASCO (Taipei, Taiwan). All animal experiments and care procedures were approved by the Institutional Animal Care and Use Committee of the Cathay General Hospital (Taipei, Taiwan; approval number: CGH-IACUC-111-030). Before undergoing the experimental procedures, all animals were physiologically normal and free from apparent infection, inflammation or neurological deficits.

### 2.2. Experimental Designs and Grouping of the Animals

For the study of the age-dependent difference in TBI-severity, adult mice between 9–12 weeks old (adult group) and middle-aged mice between 9–12 months old (aged group) were used. For the experiments of hypothyroidism in TBI-severity, adult mice between 9–12 weeks old with surgical removal of bilateral lobes of thyroid glands (Tx group) and a sham-operated (sham group) were used. For the drug treatment experiments, middle-aged mice between 9–12 months old were administered with isovolumetric solvent vehicle (1% DMSO, vehicle group), melatonin (10 mg/kg, intraperitoneal injection, melatonin group), or liothyronine sodium (10 mg/kg, intravenous injection, T3 group) at one, twenty-four, and forty-eight hours after CCI, respectively.

For the behavioral analyses, eight to ten mice for each experimental group were first subjected to a corner test, NSS score measurement, object recognition test and Y maze task two to three days before the CCI surgery. Functional outcomes were evaluated at one, three, and seven days after CCI challenge. Animals were sacrificed after the behavioral analyses to obtain the brain slices for Nissl staining.

Another cohort of animals (5 mice for each experimental group) were sacrificed at twenty-four hours after CCI challenge for biochemical analyses which included immunofluorescent staining (FJC, IBA1, GFAP, 4-HNE and 8-oxdG), measurement of MDA and hydroxyl free radicals and gene expression analyses by real-time PCR.

For the experiments with *Pearson’s* correlation analyses, an independent cohort of animals (10 from adult and 10 from aged) were scarified after the behavioral tests at three days post-CCI challenge. The individual thyroid weight, serum T3 and serum T4 were measured and associatively correlated to different behavioral (NSS and object recognition test) or biochemical (MDA and hydroxyl free radicals) indexes.

### 2.3. Animal Model of Traumatic Brain Injury

A CCI (controlled cortical impact) device was used to induce TBI, and the trauma challenge procedure was performed as described [36] with slight modifications. In brief, upon anesthesia (isoflurane, induced at 4% and maintained at 2% in air), the mouse was positioned on a stereotaxic frame. After the scalp and fascia were retracted, a craniotomy was performed to remove the skull in the shape of a circle with 3.5 mm in diameter on the right cerebral hemisphere (center of the circle: 2.0 mm lateral to the sagittal suture and 2.0 mm posterior to the bregma) to expose the dura. For the TBI impact, a 3-mm-flat impactor tip was used to impact the exposed dura at a velocity of 5 m/s, depth of 1 mm, and dwell time of 300 ms. After the brain trauma was performed, the scalp incision was sutured, and the mouse was placed on a heating pad until it had recovered from anesthesia.

### 2.4. Surgical Thyroidectomy

The surgical procedure for removal of the bilateral thyroid lobes of the mouse was performed [37]. After the animal was anesthetized by isoflurane, a vertical anterior neck incision was made and gently separated the parotid glands. After the removal of the transparent investing fascia upon the strap muscle, bilateral strap muscles were retracted laterally and held by non-traumatic forceps. Thyroid glands were then exposed and visually located between the cricoid cartilage and the first four tracheal rings. The exposed bilateral thyroid lobes were smoothly teased away from the trachea. Then, sternothyroid muscles were gently picked up with forceps, and the bilateral thyroid upper poles were removed by micro-dissecting forceps, and the recurrent laryngeal nerve (runs below the dorsal edge of the thyroid lobes) was preserved. After erasing the blood clot and rinsing with PBS, the musculature, lymphatic tissue and salivary glands were repositioned, and the surgical field was sutured. The mouse was placed on a heating pad until it recovered from anesthesia. After surgery, the mice were observed for two weeks to examine the concentration of thyroxin and triiodothyronine and subjected to TBI experiments.

### 2.5. Neurological Severity Scores

A neurological examination was performed on each mouse immediately before and at different time points after CCI challenge. The neurological severity scores (NSS), which includes a combination of motor (muscle status and abnormal movements), sensory (visual, tactile and proprioceptive), reflex and balance tests, was conducted to reflect the proper performance in motor function [36] and was calculated by using an 18-point sliding scale (normal score, 0; maximal deficit score, 18) [38]. During the NSS test, 1 point represents the inability to perform the test or the lack of a tested reflex. Therefore, a higher score indicates higher levels of severity.

### 2.6. Object Location Recognition Test

To assess hippocampal-mediated recognition memory, we conducted the novel location recognition test [39,40]. The experimental apparatus for the test was composed of a white-color box, 50 × 50 × 50 cm^3^ in size and placed in the center of a dimmed light-illuminated room. During the habituation session, the animals were allowed to explore the whole arena of the box without any testing objects for 20 min. Various visual cues were attached to the walls of the testing apparatus to provide contextual guiding information. One hour after the habituation session, the mouse will be replaced back to the testing apparatus and allowed to explore two identical adjacently located objects for 10 min and returned to their home cage. One hour later, a testing trial was performed by repositioning one of the objects to another corner of the testing apparatus. The testing session consisted of a 10 min exploration of the two objects, and the time the mouse spent in exploring each object was recorded using a digital video camera and scoring the preference for the repositioned object by the behavioral tracking system Ethovision XT (Noldus). A discrimination index was calculated by the formula of [time spent on the object in novel location/(time spent on the object in novel location + time spent on the object in familiar location)] to reflect the cognitive performance.

### 2.7. Spontaneous Alteration in Y-Maze Testing

The spontaneous alternation Y-maze has long been applied for the assessment of short-term spatial working memory [41,42]. In our current study, a Y shaped maze with visual cues on the wall of different arms was used as a testing apparatus. The test was executed in the dimmed, light-illuminated room, and mice were placed in the center of the Y-maze as a starting point for the test. Mice were allowed to freely explore the Y-maze for 10 min, and the sequential order of each arm visit was video-recorded and analyzed for the spontaneous alternation behavior in their exploration. Each arm visit was defined as the mouse body center moving into the specific arm, and the alteration was defined as the consecutive entry into three different arms which was calculated as the percentage of alteration: total number of alternations/maximum possible alternations (total number of arms entered-2) × 100%.

### 2.8. Corner Test

As the damage in the striatal or cortical area might cause severe sensory and motor asymmetries in postural biases, limb usage, and tactile hairs function, the corner test has been increasingly applied in murine brain injury studies to evaluate the sensory-motor asymmetry [43]. The testing apparatus is comprised of two connected nontransparent walls (25 × 15 cm) which form a gap with a 30-degree angle. The trial was conducted before and at different time points after the CCI challenge by placing the animal halfway into the corner and facing the wall junction. When mice walked into the corner and the vibrissae touched the wall, the initiation of the turning behavior (rear and turn to the left or right side) was recorded. A successful turning must include rearing movement and forelimbs touched down to the floor. The asymmetrical preference in turning was analyzed after conducting ten successful turns in the left or right to define the laterality index.

### 2.9. T_4_ and T_3_ Measurements

ELISA kits (T4044T-100 and T3043T-100; Calbiotech, El Cajon, CA, USA) were used to determine the concentration of free thyroxin (T_4_) and free triiodothyronine (T_3_) from the serum sample of the testing mice. In brief, the blood specimens of animals with/without CCI challenge were collected by syringe (with EDTA) via the inferior vena cava. The specimens were then centrifuged at room temperature with a speed of 300× *g* for 10 min to obtain the serum samples. The assay procedures followed the manufacturer’s instructions, and the absorbance of the mixtures was measured at 450 nm on a microplate reader.

### 2.10. MDA Measurements

Malondialdehyde (MDA) is a highly reactive three-carbon dialdehyde produced as a byproduct during the process of polyunsaturated fatty acid peroxidation that has been commonly used as a reliable biomarker of lipid peroxidation and oxidative stress. Following the manufacturer’s instructions (10,009,055, Cayman Chemical, Ann Arbor, MI, USA), equal amounts (approximately 25 mg) of injured brain tissue blocks were homogenized and centrifuged at 1600× *g* for 10 min at 4 °C. The supernatants were applied for performing the MDA measurements at the absorbance of 535 nm.

### 2.11. Measurement of Hydroxyl Free Radicals

The concentration of hydroxyl free radicals was determined by using the hydroxyl free radical (-OH) colorimetric assay kit (MBS2540424, Mybiosource, San Diego, CA, USA). The free prepared brain tissue blocks were subjected to analysis of the production of hydroxyl free radicals following the manufacturer’s instructions. The O.D. value of the final product solution was measured at 550 nm by a microplate reader.

### 2.12. Immunofluorescent Staining

The mice were sacrificed after the CCI challenge based on different experimental designs. Mice were deeply anesthetized with the air mixture containing 75% air and 5% isoflurane maintained in 25% of oxygen and perfused transcardially with cold phosphate-buffered solution followed by 4% formaldehyde (Sigma-Aldrich, Merck, St. Louis, MO, USA). After perfusion, the brain was removed and immersion-fixed in 4% formaldehyde solution overnight. After fixation, the whole brain was immersed in 30% sucrose solution for more than three days. The brain sections in 50 µm were prepared by a sliding Microtome (Leica, Nussloch, Germany) and processed for immunofluorescence staining. At the beginning, the brain sections were immersed in PBS containing 6% donkey serum and 0.25% Triton X-100 for blocking of the non-specific antibody binding. Then, brain sections were incubated with the mixture of primary antibodies diluted with SignalPlus antibody enhancer solution 1 (GeneTex, Irvine, CA, USA, cat# 49999) overnight at 4 °C. The antibodies and the concentration used are listed below: GFAP (1:1000; Abcam, ab4674), IBA-1 (1:1000; Abcam, ab4674), 8-oxo-dG (1:200; JaICA, MOG-100P), NeuN (1:1000; Abcam, ab177487) and 4-Hydroxynonenal (1:1000; Abcam, ab46545). Brain sections were then incubated with a mixture of secondary antibodies CF^®^488A, CF^®^568 and CF^®^633 (Biotium, Hayward, CA, USA) and diluted with SignalPlus enhancer solution 2 (GeneTex, Irvine, CA, USA, Cat# 49999) overnight at 4 °C. After washing three times in PBS containing 0.1% tween 20, the brain sections were mounted and coverslipped with ProLong™ glass antifade mountant (Invitrogen™, Thermo Fisher Scientific, Waltham, MA, USA, P36980). All immunofluorescencent images were obtained from brain sections taken by Leica STELLARIS 8 (Leica Microsystems) with the 40 × objectives. The 8-oxo-dG/NeuN double-positive neurons, 4-Hydroxynonenal/NeuN double-positive neurons, IBA-1 positive microglia and GFAP positive astrocyte in the CA1 regions of the hippocampus were manually counted in three predetermined 50 × 50 µm squares per image and presented as the number of positive neurons per mm^2^.

### 2.13. Real-Time RT-PCR Analysis

Mice from different experimental groups were sacrificed after CCI challenge. Mice were deeply anesthetized with the air mixture containing 75% air and 5% isoflurane maintained in 25% of oxygen and perfused transcardially with cold phosphate-buffered solution. The hippocampal tissue blocks were immediately harvested on an ice-cold metal plate, snap-frozen in liquid nitrogen, homogenized and RNA extracted using the Qiagen RNeasy Plus Mini Kit (Qiagen, Valencia, CA, USA) and stored at −80 °C. One μg of total RNA was reverse transcribed to cDNA using the High-Capacity cDNA Reverse Transcription Kit (Applied Biosystems™, Thermo Fisher Scientific, 4368814). Samples were quantified using QuantiNova PCR Kits (Qiagen, Hilden, 208252) by using the StepOnePlus™ Real-Time PCR System (Applied Biosystems™, Thermo Fisher Scientific,). The nucleotide sequences of the primers used for amplification were as follows: for CAT (NM_009804), sense 5′-CGGCA CATGA ATGGC TATGG ATC-3′ and antisense 5′-AAGCC TTCCT GCCTC TCCAA CA-3′; for SOD1 (NM_011434), sense 5′-GGTGA ACCAG TTGTG TTGTC AGG-3′ and antisense 5′- ATGAG GTCCT GCACT GGTAC AG-3′; for SOD2 (NM_013671), sense 5′-TAACG CGCAG ATCAT GCAGC TG-3′ and antisense 5′-AGGCT GAAGA GCGAC CTGAG TT-3′; for GPX1 (NM_008160), sense 5′-CGCTC TTTAC CTTCC TGCGG AA-3′ and antisense 5′-AGTTC CAGGC AATGT CGTTG CG-3′; for TNF (NM_013693), sense 5′- GGTGC CTATG TCTCA GCCTC TT-3′ and antisense 5′-GCCAT AGAAC TGATG AGAGG GAG-3′; for IL-6 (NM_031168), sense 5′-TACCA CTTCA CAAGT CGGAG GC-3′ and antisense 5′-CTGCA AGTGC ATCAT CGTTG TTC-3′; for IL-1B (NM_008361), sense 5′- TGGAC CTTCC AGGAT GAGGA CA-3′ and antisense 5′-GTTCA TCTCG GAGCC TGTAG TG-3′; for CXCL1 (NM_008176), sense 5′-TCCAG AGCTT GAAGG TGTTG CC-3′ and antisense 5′- AACCA AGGGA GCTTC AGGGT CA-3′; for BCL2 (NM_009741), sense 5′-CCTGT GGATG ACTGA GTACC TG-3′ and antisense 5′-AGCCA GGAGA AATCA AACAG AGG-3′; for BCL2L1 (NM_009743), sense 5′-GCCAC CTATC TGAAT GACCA CC-3′ and antisense 5′-AGGAA CCAGC GGTTG AAGCG C-3′; for Bax (NM_007527), sense 5′ AGGAT GCGTC CACCA AGAAG CT-3′ and antisense 5′-TCCGT GTCCA CGTCA GCAAT CA-3′; for BAK1 (NM_007523), sense 5′-GGAAT GCCTA CGAAC TCTTC ACC-3′ and antisense 5′-CAAAC CACGC TGGTA GACGT AC-3′; for GAPDH (NM_008084), sense 5′-GAACA TCATC CCTGC ATCCA -3′ and antisense 5′-GCCAG TGAGC TTCCC GTTCA -3′.

### 2.14. Fluoro-Jade C (FJC) Staining

Fluoro-Jade C (FJC) staining has been widely used for the specific detection of degenerating neurons undergoing either apoptotic, necrotic or pyroptotic neuronal death. Since recent evidence indicated that FJC could also label degenerating non-neuronal cells [44], we also conducted double staining for the neuronal marker NeuN to faithfully identify the degenerating neurons. The brain sections were processed for FJC staining according to the Fluro-Jade^®^ C RTDTM stain reagent user manual. Briefly, 50 µm brain tissue sections were mounted on the silane-coated slide (5116, Muto Kagaku, Tokyo, Japan) and air dried with slide warmer at 50 °C for 30 min. Following rehydration for 2 min in distilled water, the brain sections were immersed with potassium permanganate for 5 min after being rinsed for 2 min in fresh distilled water. The slices were then incubated in FJC and DAPI for 25 min in the dark. The slides were washed three times in distilled water and left to dry on a slide warmer at 50 to 60 °C. The dried brain sections were then cleared by immersion in xylene for 5 min and coverslipped with DPX mountant for histology (Sigma-Aldrich, Merck, USA, 06522-100 mL). A Leica STELLARIS 8 (Leica Microsystems, Germany) was used to examine the FJC-stained sections (40× objective with a zooming factor of 1.5). The FJC and NeuN double-positive neurons in the CA1 regions of the hippocampus were manually counted in three predetermined 50 × 50 µm squares per image and presented as the number of FJC positive neurons per mm^2^.

### 2.15. Nissl Staining

The histological method devised by Nissl is widely used to study neuronal morphology and pathology [45]. Brain sections 50 µm thick were mounted onto silane-coated slides (5116, Muto Kagaku, Japan), air-dried and covered with slide warmers at 50 °C for 30 min. Sections mounted on slides were thoroughly dried and rehydrated with distilled water for 2 min. The slides were then dipped in cresyl violet stain solution (1%, Abcam, UK, ab246817) for 5 min, followed by immersion in a solution of acetic acid in 95% ethanol (1:50,000 dilution) for 5 min. Brain sections were dehydrated by immersion in 100% ethanol for 1 min, treated with xylene for 10 min and coverslipped with DPX mountant for histological analyses (Sigma-Aldrich, Merck, St. USA, 06522-100 mL). Nissl stained slides were scanned on an Olympus CX33 (Olympus Corporation, Japan) using a manual WSI whole slide scanner (Microvisioneer, Esslingen am Neckar, Germany) with 40× magnification. The neuronal density in the CA1 regions of the hippocampus was manually counted in three predetermined 50 × 50 µm squares per image and represented as the number of surviving neurons per mm^2^.

### 2.16. Statistical Analysis

All the results are presented as mean ± SEM. The analyses for a significant difference between two experimental groups were calculated by using the two-tailed unpaired student *t*-test. The comparison between multiple groups with more than two were analyzed with a one-way analysis of variance (ANOVA) followed by Bonferroni’s post hoc analyses for assessing the significance between any of the isolated experimental groups. *Pearson’s* correlation analysis was performed for the investigation of the correlation analyses between two parameters. Probability values of *p* < 0.05 were considered to represent statistical significance. All the statistical analyses were performed by using the Prism 6 (GraphPad) software.

## 3. Results

### 3.1. Age-Dependent Difference in TBI Susceptibility

**Figure 1:**
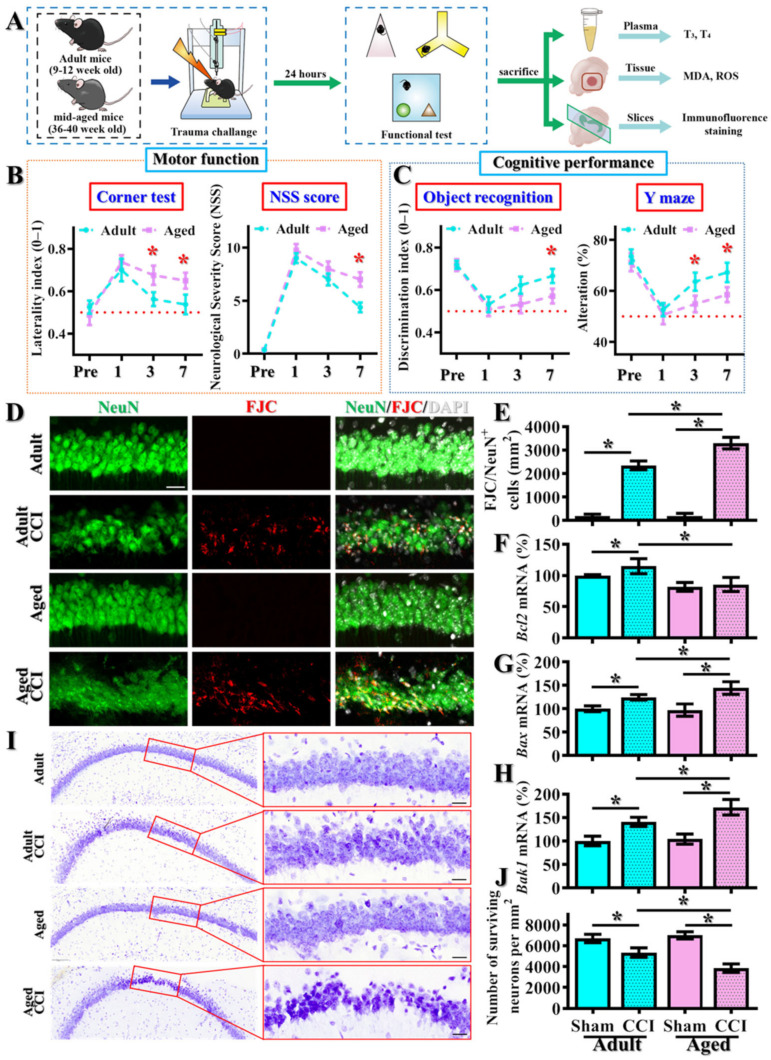
Increase in TBI-susceptible phenotypes in the middle-aged animals. (**A**) Schematic illustration of experimental designs; (**B**) Behavioral performance of motor function, including corner test and neurological severity scores at different time points after CCI challenge; (**C**) Behavioral performance of cognitive function in an object location recognition task and spontaneous alternation Y maze. Data are presented as mean ± SEM. *n* = 8 to 10 mice; (**D**,**E**) Quantification and representative images of FJC-positive degenerating neurons. *n* = 15 images from 5 mice; (**F**–**H**) Quantification of the relative expression levels of *Bcl2*, *Bax* and *Bak1* mRNAs from different experimental groups. *n* = 5 mice; (**I**,**J**) Quantification and representative images of Nissl stains showing the surviving neurons. *n* = 15 images from 5 mice. Data are presented as mean ± SEM in (**E**–**J**) and analyzed by a one-way ANOVA with Bonferroni’s post hoc analysis. * *p* < 0.05. Scale = 25μm.

By using the standard rodent model of TBI and controlled cortical impact (CCI), we first evaluated if there might be an age-dependent difference in TBI susceptibility. Both the functional recovery of motor (corner test and NSS scores) and cognitive performance (novel object location task and spontaneous alternation in Y maze) were evaluated at the time points before CCI, and one, three and seven days after CCI (Figure 1A). We found that the CCI challenge resulted in similar levels of behavioral deficits in both adult and middle-aged animals at one day after brain trauma (Figure 1B,C). Interestingly, as the behavioral deficits gradually recovered at three and seven days after CCI in the adult group, we found a significant impairment in functional recovery of both motor (Figure 1B) and cognitive performance (Figure 1C) in the middle-aged group of animals. Basically, we observed an increase in asymmetrical turning tendency in the corner test, higher NSS scores, reduced discrimination in a novel object task and alternation in arm choice in the middle-aged animals.

The observation of impaired functional recovery after CCI in the aged animals raised the possibility of increasing in neuronal damage in these susceptible individuals. By FJC staining, we found that CCI challenge significantly increased FJC-positive degenerating neurons twenty-four hours after the brain trauma (Figure 1D,E, adult/CCI, 2350 ± 191.8 cells per mm^2^ vs. adult/sham, 200 ± 75.6 cells per mm^2^, *p* < 0.01). Remarkably, the CCI challenge induced even higher numbers of FJC-positive degenerating neurons in the middle-aged animals (Figure 1D,E, aged/CCI, 3300 ± 247.8 cells per mm^2^ vs. adult/CCI, 2350 ± 191.8 cells per mm^2^, *p* = 0.0028). Meanwhile, real-time PCR analyses indicate the suppression of the expression of anti-apoptotic molecule *Bcl2* (Figure 1F) and profoundly enhanced the expression of pro-apoptotic molecules *Bax* and *Bak1* (Figure 1G,H) at twenty-four hours after CCI challenge in the middle-aged animals.

Furthermore, we also tested if the enhanced pro-apoptotic events and increased degenerating neurons at the acute stage (twenty-four hours after CCI) could eventually result in the loss of surviving neurons at later advanced stages (one week after CCI) in the middle-aged animals. By using Nissl staining to visualize the surviving neurons, we found that CCI challenge significantly reduced the number of surviving neurons in the hippocampal CA1 in the adult animals (Figure 1I,J, adult/CCI, 5350 ± 446.8 cells per mm^2^ vs. adult/sham, 6700 ± 398.2 cells per mm^2^, *p* < 0.05). In the meantime, the CCI challenge induced an even more dramatic decrease of surviving neurons in the middle-aged animals (Figure 1I,J, aged/CCI, 3850 ± 413.6 cells per mm^2^ vs. adult/CCI, 5350 ± 446.8 cells per mm^2^, *p* < 0.05).

**Figure 2:**
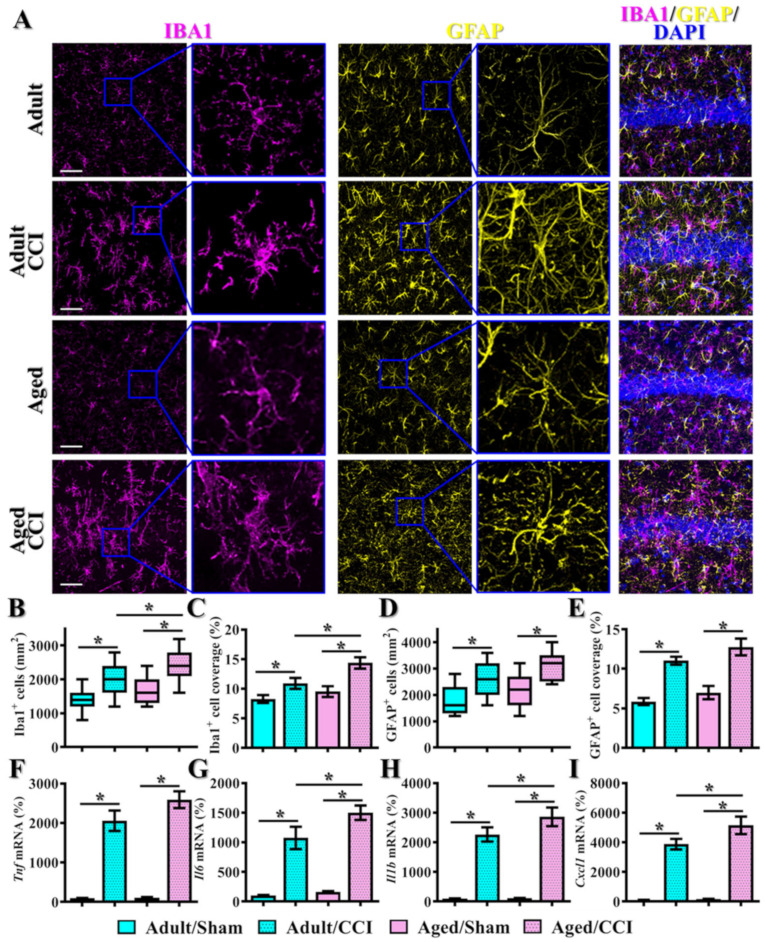
Increase in microgliosis and astrogliosis after CCI in the middle-aged animals. (**A**) Representative images of IBA1-positive microglial cells (color in magenta) and GFAP-positive astroglial cells (color in yellow) from different experimental groups. Blue, DAPI signal for the nuclear stain. Scale = 25μm; (**B**,**C**) Quantification of the regional density and percentage of area covered by IBA1 positive cells; (**D**,**E**) Quantification of the regional density and percentage of area covered by GFAP positive cells. *n* = 15 images from 5 mice in (**B**–**E**); (**F**–**I**) Quantification of the relative expression levels of *Tnf*, *Il6, Il1b* and *Cxcl1* mRNAs from different experimental groups. *n* = 5 mice. All the data are presented as mean ± SEM and analyzed by a one-way ANOVA with Bonferroni’s post hoc analysis. * *p* < 0.05.

Exaggerated inflammatory responses elicited by reactive microgliosis or astrogliosis have been correlated to aberrant neuronal damage and impaired functional recovery after TBI [46,47]. Therefore, we further examined if there might be an age-dependent difference in the phenotypes of microgliosis or astrogliosis after brain trauma. Through the staining of IBA1 (ionized calcium-binding adapter molecule 1) for the labeling of microglial cells, we found that CCI challenge resulted in the morphological activation of microglial cells from a highly ramified structure into the amoeboid cell shape with a larger cell body and thicker processes (Figure 2A). Remarkably, the phenotype of increased microgliosis by CCI is further exaggerated in the middle-aged animals, as reflected by the increased regional density of microglial cells (Figure 2B, aged/CCI, 2489 ± 160.2 cells per mm^2^ vs. adult/CCI, 2040 ± 151.4 cells per mm^2^, *p* < 0.05) and percentage of area covered by IBA1 positive cells (Figure 2C, aged/CCI, 14.36 ± 0.973% vs. adult/CCI, 10.89 ± 0.926%, *p* < 0.05).

Meanwhile, staining with the GFAP (glial fibrillary acidic protein) antibody was conducted for the labeling of astroglial cells. As demonstrated in Figure 2A, the CCI challenge resulted in significant morphological activation of astroglial cells with the thicker and highly elaborated processes (Figure 2A). Notably, we found CCI-induced astrogliosis is profoundly enhanced in the middle-aged animals, as indexed by an increased regional density of GFAP positive cells (Figure 2B, aged/CCI, 3100 ± 196.4 cells per mm^2^ vs. adult/CCI, 2600 ± 250.7 cells per mm^2^, *p* < 0.05) and percentage of area covered by GFAP positive cells (Figure 2C, aged/CCI, 12.97 ± 0.871% vs. adult/CCI, 10.7 ± 0.447%, *p* < 0.05). Meanwhile, real-time PCR analyses indicate the expression levels of different proinflammatory cytokines and chemokines such as *Tnf, Il6*, Il1b and *Cxcl1* were significantly increased after CCI challenge in the middle-aged animals (Figure 2F–I). Combined with all the behavioral, morphological and biochemical analyses, we first confirm the presence of age-dependent differences in TBI susceptibility.

### 3.2. Hypothyroidism Links to TBI-Susceptible Phenotypes

**Figure 3:**
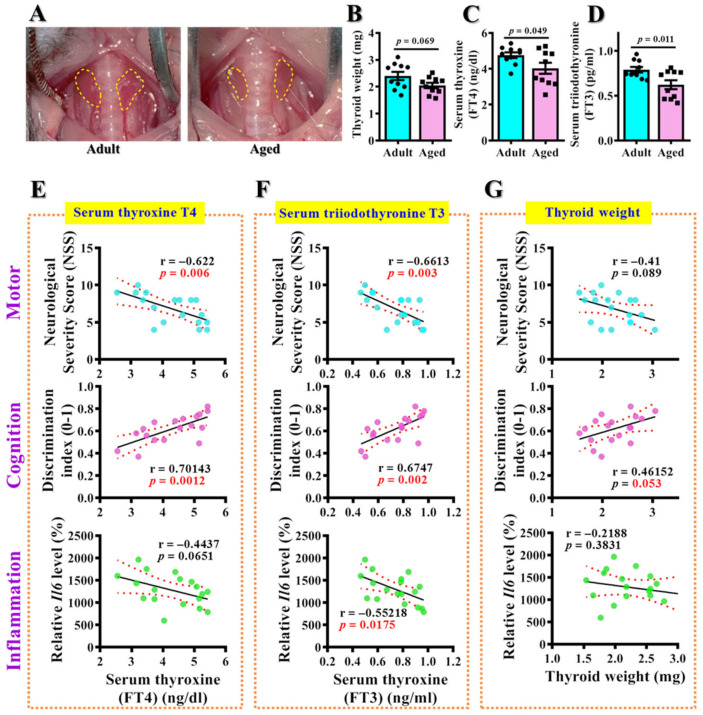
**Correlation of thyroid function to the TBI-susceptible phenotypes.** (**A**) Representative images showing the anatomical outlines of bilateral thyroid lobes from adult and middle-aged animals; (**B**–**D**) Quantification of the thyroid weight, serum free T4 and free T3 levels. *n* = 8 to 10 mice. Data are presented as mean ± SEM and are analyzed by a two-tailed unpaired *t*-test. (**E**) *Pearson’s* correlation plots between individual serum T4 with their behavioral or inflammatory indexes after CCI; (**F**) *Pearson’s* correlation plots between individual serum T3 with their behavioral or inflammatory indexes after CCI; (**G**) *Pearson’s* correlation plots between individual thyroid weight with their behavioral or inflammatory indexes after CCI. *n* = 20 mice.

Accumulating evidence indicates an increased prevalence of thyroid dysfunction in the aged population. Thus, we first tested if the observed TBI-susceptible phenotypes in the middle-aged animals might be correlated to an individual’s thyroid function. Experimentally, we sacrificed the adult or middle-aged animals at three days after CCI challenge. Then, two lobes of thyroid glands (Figure 3A), serum levels of free form T4 (thyroxine) and free form T3 (triiodothyronine) were collected and measured to index the changes in thyroid function. We found there is a significant decrease in thyroid function in middle-aged animals (Figure 3A–D). The middle-aged animals displayed a mild reduction in thyroid weight (Figure 3B), a decrease in serum T4 (Figure 3B) and a significant decrease in serum T3 levels (Figure 3C) compared to the adult animals.

To answer if the observed TBI-susceptible phenotypes in the middle-aged animals might correlate with the decrease in thyroid function, we further conducted the *Pearson’s* correlation analyses to understand the role of the thyroid gland with different TBI phenotypes. We found that both serum levels of free T4 (Figure 3E) and free T3 (Figure 3F) significantly correlated to the increased neurological severity scores, the impaired discrimination in novel object location test and enhanced production of the inflammatory molecule Il6 after TBI. A relatively mild correlation was also observed in thyroid weight to the motor, cognition and inflammatory indexes (Figure 3G). These findings strongly suggested a possible role of changes in thyroid function that contribute to the TBI-susceptible phenotypes in aged animals.

**Figure 4:**
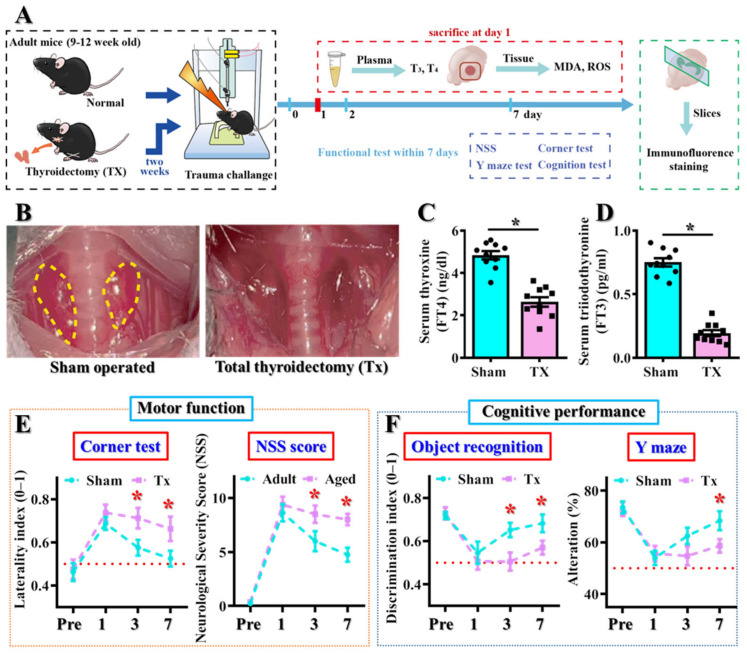
**Increase in TBI-susceptible phenotypes after surgical thyroidectomy.** (**A**) Schematic illustration of the experimental designs; (**B**) Representative images showing the surgical removal of bilateral thyroid lobes. (**C**,**D**) Quantification of the serum free T4 and free T3 levels. *n* = 8 to 10 mice. Data are presented as mean ± SEM and are analyzed by a two-tailed unpaired *t*-test. * *p* < 0.05. (**E**) Behavioral performance of motor function, including corner test and neurological severity scores at different time points after CCI challenge; (**F**) Behavioral performance of cognitive function in the object location recognition task and spontaneous alternation Y maze at different time points after CCI. *n* = 8 to 10 mice. Data are presented as mean ± SEM and are analyzed by a one-way repeated measures ANOVA with Bonferroni’s post hoc analysis. * *p* < 0.05.

To provide direct evidence for the pathological role of thyroid dysfunction in modulating individual TBI-susceptibility, we conducted a total thyroidectomy (Tx) in the adult (9–12 weeks of age) animals to answer if the loss of thyroid function could lead to a TBI-susceptible phenotype (Figure 4A). Experimentally, the adult mice had surgical removal of bilateral lobes of the thyroid glands (Figure 4B) and recovered for two weeks before CCI challenge. We first confirmed if the removal of thyroid glands could result in hypothyroidism of these animals. We found that both serum levels of T4 (Figure 4C) and T3 (Figure 4D) decreased significantly two weeks after Tx surgery. Then, both motor and cognitive behavioral analyses were conducted to evaluate the impact of thyroid dysfunction on CCI-susceptibility (Figure 4A).

The CCI challenge resulted in similar levels of behavioral deficits in both sham-operated and thyroidectomized animals at one day after brain trauma (Figure 4E,F) which indicates that the surgical procedure of thyroidectomy does not affect the TBI severity at acute stages after the insult. Remarkably, as the behavioral deficits in motor and cognitive performance gradually recovered at three and seven days after CCI in the sham-operated animals, there is an impairment of functional recovery of both motor (Figure 4E) and cognitive performance (Figure 4F) in the thyroidectomized animals. We found a significantly higher asymmetrical turning tendency in the corner test, an increase in NSS scores, poorer discrimination in novel object test and a reduction in the alternation of arm choice in the Tx animals.

**Figure 5:**
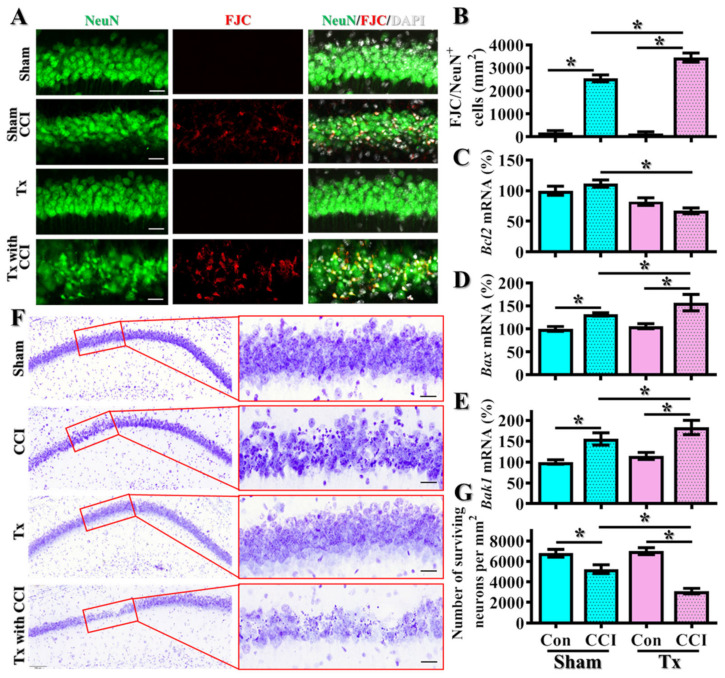
**Increase in TBI-induced neuronal loss after surgical thyroidectomy.** (**A**,**B**) Quantification and representative images of FJC-positive degenerating neurons in the hippocampus. *n* = 15 images from 5 mice; (**C**–**E**) Quantification of the relative expression levels of *Bcl2*, *Bax* and *Bak1* mRNAs from different experimental groups. *n* = 5 mice. (**F**,**G**) Quantification and representative images of Nissl stains showing the surviving neurons in the hippocampus. *n* = 15 images from 5 mice. Data are presented as mean ± SEM (**B**–**G**) and analyzed by a one-way ANOVA with Bonferroni’s post hoc analysis. * *p* < 0.05. Scale = 25 μm.

Since we found the surgical removal of thyroid glands recapitulates the TBI-susceptible phenotypes similar to what we observed in the middle-aged animals, we also tried to confirm if there might be an increase in neurodegeneration. Through FJC staining, we found that CCI challenge significantly increased the FJC-positive degenerating neurons in sham-operated animals after TBI (Figure 5A,B, sham/CCI, 2550 ± 150 cells per mm^2^ vs. sham/con, 222 ± 70.3 cells per mm^2^, *p* < 0.01). Importantly, the CCI challenge resulted in significantly higher numbers of FJC-positive degenerating neurons in the thyroidectomized animals (Figure 5A,B, Tx/CCI, 3450 ± 199.1 cells per mm^2^ vs. sham/CCI, 2550 ± 150 cells per mm^2^, *p* < 0.01). Furthermore, real-time PCR analyses show a profound decrease in the expression levels of the anti-apoptotic molecule *Bcl2* (Figure 5C); however, expression levels significantly increased in the pro-apoptotic molecules *Bax* and *Bak1* (Figure 5D,E) after CCI challenge in the thyroidectomized animals. As demonstrated in Figure 5F,G, CCI challenge significantly decreased the number of surviving neurons in the hippocampal CA1 region in sham-operated animals (Figure 5F,G, sham/CCI, 5250 ± 430.5 cells per mm^2^ vs. sham/con, 6800 ± 370.3 cells per mm^2^, *p* = 0.0275). Remarkably, surgical removal of the bilateral thyroid glands significantly aggravated the CCI challenge-induced loss of surviving neurons in the hippocampus (Figure 5F,G, Tx/CCI, 3111 ± 264.8 cells per mm^2^ vs. sham/CCI, 5250 ± 430.5 cells per mm^2^, *p* < 0.01).

**Figure 6:**
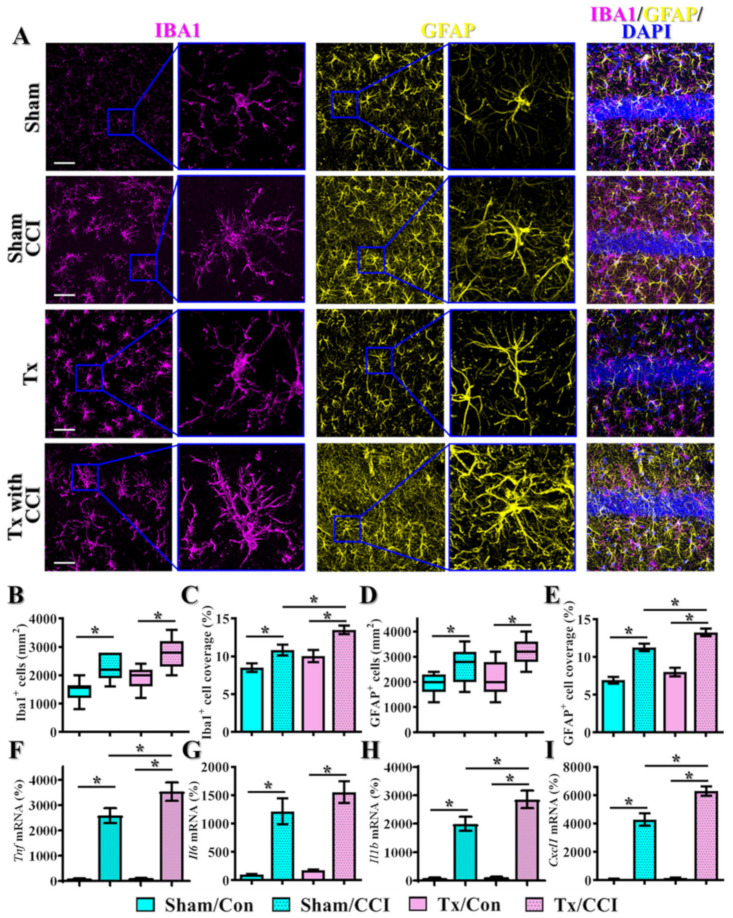
**Increase in microgliosis and astrogliosis after CCI in the thyroidectomized animals.** (**A**) Representative images of IBA1-positive microglial cells (color in magenta) and GFAP-positive astroglial cells (color in yellow) from different experimental groups. Blue, DAPI signal for the nuclear stain. Scale = 25μm; (**B**,**C**) Quantification of the regional density and percentage of area covered by IBA1-positive cells; (**D**,**E**) Quantification of the regional density and percentage of area covered by GFAP-positive cells. *n* = 15 images from 5 mice in (**B**–**E**). (**F**–**I**) Quantification of the relative expression levels of *Tnf*, *Il6, Il1b* and *Cxcl1* mRNAs from different experimental groups with sham-operated or surgical removal of thyroid glands. *n* = 5 mice. All the data are presented as mean ± SEM and are analyzed by a one-way ANOVA with Bonferroni’s post hoc analysis. * *p* < 0.05.

In addition to the observed TBI-susceptible phenotypes of behavioral impairment and neurodegeneration in thyroidectomized animals, we also evaluated if there might be exaggerated inflammatory responses. We found that the CCI challenge resulted in the morphological activation of microglial cells into the reactive amoeboid cell shape in both sham-operated and thyroidectomized animals (Figure 6A). Notably, the levels of increased microgliosis by CCI challenge are significantly enhanced in the thyroidectomized animals. Both the regional density of microglial cells (Figure 6B, Tx/CCI, 2800 ± 168.7 cells per mm^2^ vs. sham/CCI, 2240 ± 148.5 cells per mm^2^, *p* = 0.05) and percentage of area covered by IBA1 positive cells (Figure 6C, Tx/CCI, 13.5 ± 0.575% vs. sham/CCI, 10.83 ± 0.715%, *p* < 0.05) were profoundly exaggerated in the thyroidectomized animals. In addition, the phenomenon of astrogliosis was significantly enhanced by the CCI challenge in both sham-operated and thyroidectomized animals (Figure 6A). Remarkably, we found CCI-induced astrogliosis is profoundly enhanced in the thyroidectomized animals in which we observed a mild but not a significant increase in regional density of GFAP positive cells (Figure 6B, Tx/CCI, 3156 ± 169.2 cells per mm^2^ vs. sham/CCI, 2622 ± 222.2 cells per mm^2^, *p* = 0.353) and a significant increase in the percentage of area covered by GFAP-positive cells (Figure 6C, Tx/CCI, 13.26 ± 0.502% vs. sham/CCI, 11.26 ± 0.496%, *p* = 0.05). Accordingly, gene expression analyses of the alteration in proinflammatory cytokines and chemokines by real-time PCR analyses indicate that there is a significant increase in the expression of *Tnf, Il6, Il1b* and *Cxcl1* after CCI challenge in the thyroidectomized animals (Figure 6F–I). Combining all the behavioral, morphological and biochemical analyses with surgical removal of thyroid glands strongly suggests that thyroid dysfunction pathologically contributes to the observed TBI-susceptible phenotypes.

### 3.3. Excessive Oxidative Stress Links to the TBI-Susceptible Phenotypes

As the functional significance of thyroid hormone in oxidative regulation is reported, it could be interesting to explore if the observed TBI-susceptible phenotypes in middle-aged and thyroidectomized individuals might be associated with exaggerated oxidative stress in the brain after CCI challenge.

**Figure 7:**
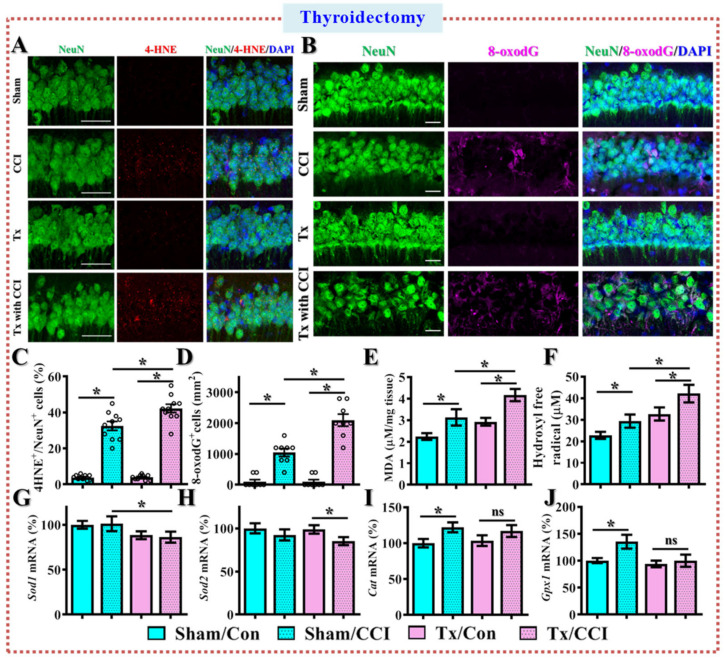
**Increase in oxidative stress after CCI in the thyroidectomized animals.** (**A**) Representative images of 4-HNE-positive (color in red) neurons (NeuN, color in green) from different experimental groups; (**B**) Representative images of 8-oxodG-positive (color in magenta) neurons (NeuN, color in green). Blue, DAPI signal for the nuclear stain. Scale = 25μm; (**C**) Quantification of the percentage of neurons with 4-HNE-positive puncta; (**D**) Quantification of the regional density of the 8-oxodG-positive neurons. *n* = 15 images from 5 mice in (**C**) and (**D**); (**E**) Quantification of the levels of Malondialdehyde; (**F**) Quantification of the production of hydroxyl free radicals; (**G**–**J**) Quantification of the relative expression levels of *Sod1*, *Sod2*, *Cat* and *Gpx1* mRNAs from different experimental groups with sham-operated or surgical removal of thyroid glands. *n* = 5 mice. All the data are presented as mean ± SEM and analyzed by a one-way ANOVA with Bonferroni’s post hoc analysis. * *p* < 0.05; ns, not significant.

By using 4-hydroxynonenal (4-HNE) staining for the level of lipid peroxidation, we found that CCI challenge significantly increased the percentage of positive-stained neurons with 4-HNE puncta in sham-operated animals after TBI (Figure 7A,C, sham/CCI, 32.5 ± 2.47% vs. sham/con, 3.778 ± 0.547%, *p* < 0.001). Remarkably, the CCI challenge resulted in an even significantly higher percentage of neurons with 4-HNE positive puncta in the thyroidectomized animals (Figure 7A,C, Tx/CCI, 42.3 ± 2.31% vs. sham/CCI, 32.5 ± 2.47%, *p* = 0.0026). By using 8-oxodG antibody for the detection of oxidative DNA damage, we found that CCI challenge significantly increased the number of positive-stained neurons with an 8-oxodG signal in sham-operated animals after TBI (Figure 7B,D, sham/CCI, 1050 ± 129.6 cells per mm^2^ vs. sham/con, 100 ± 65.47 cells per mm^2^, *p* < 0.001). Remarkably, the CCI challenge resulted in an even more significant increase in the number of 8-oxodG positive neurons in the thyroidectomized animals (Figure 7B,D, Tx/CCI, 2100 ± 196.4 cells per mm^2^ vs. sham/CCI, 1050 ± 129.6 cells per mm^2^, *p* < 0.001).

Through a quantitative analyses of MDA levels, we found that CCI induced an increase in MDA levels and is significantly higher in the thyroidectomized animals compared to sham-operated groups (Figure 7E, Tx/CCI, 4.163 ± 0.28 μM/mg tissue vs. sham/CCI, 43.125 ± 0.378 μM/mg tissue, *p* < 0.05). Meanwhile, using freshly prepared hippocampal slices, we directly measured the production of hydroxyl free radicals. As we demonstrated in Figure 7F, the CCI challenge significantly increased the synthesis of reactive oxygen species in both sham-operated and thyroidectomized animals. Remarkably, there is a significantly higher level of hydroxyl free radicals in the thyroidectomized animals after brain trauma (Figure 7F).

Since thyroid hormones have been reported to regulate the expression levels of various antioxidant genes, we then tested the mRNA levels of *Sod1, Sod2, Cat* and *Gpx1* by real-time PCR analyses. We found there is a compensatory increase in *Cat* and *Gpx1* after CCI challenge in the sham-operated animals (Figure 7G,J). Interestingly, surgical removal of thyroid glands completely abolished such a protective compensatory increase in antioxidant genes after CCI challenge in the thyroidectomized animals (Figure 7G,J). These results indicate that there might be a thyroid hormone-dependent compensatory regulation of antioxidant events, which could be obliterated after surgical thyroidectomy.

**Figure 8:**
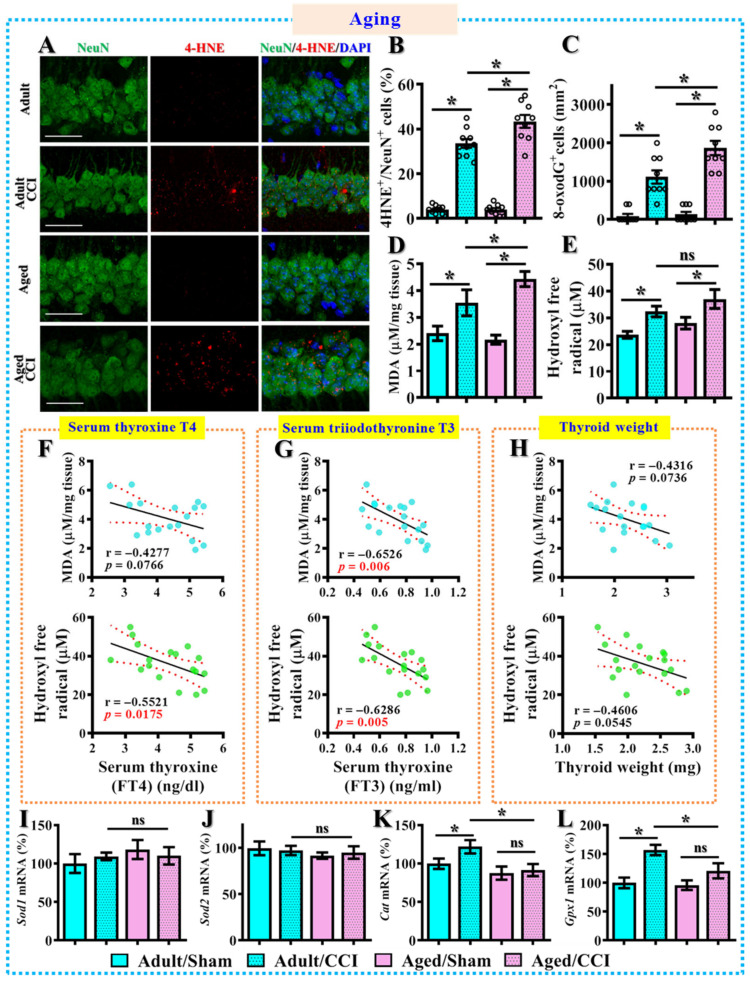
**Increase in oxidative stress after CCI in the middle-aged animals.** (**A**,**B**) Representative images and quantification analyses of 4-HNE-positive (color in red) neurons (NeuN, color in green). Scale = 25μm. *n* = 15 images from 5 mice; (**C**) Quantification of the regional density of the 8-oxodG positive neurons in the hippocampus; (**D**) Quantification of the levels of malondialdehyde; (**E**) Quantification of the production of hydroxyl free radicals. *n* = 5 mice in (**D**,**E**); (**F**) *Pearson’s* correlation plots between individual serum T4 with MDA levels or production of hydroxyl free radicals after CCI; (**G**) *Pearson’s* correlation plots between individual serum T3 with MDA levels or production of hydroxyl free radicals after CCI; (**H**) *Pearson’s* correlation plots between individual thyroid weight with MDA levels or production of hydroxyl free radicals after CCI. *n* = 20 mice in (**F**–**H**); (**I**–**L**) Quantification of the relative expression levels of *Sod1*, *Sod2, Cat* and *Gpx1* mRNAs. *n* = 5 mice. All the data are presented as mean ± SEM and are analyzed by a one-way ANOVA with Bonferroni’s post hoc analysis. * *p* < 0.05; ns, not significant.

Since we found that the surgical removal of bilateral lobes of thyroid glands recapitulates TBI-susceptible phenotypes, we then examined if there might be a similar excessive oxidative stress in the middle-aged animals. Through 4-HNE staining, we found that the CCI challenge resulted in a significantly higher percentage of neurons with 4-HNE-positive puncta in the middle-aged animals compared to the adult animals (Figure 8A,B, aged/CCI, 43.44 ± 2.911% vs. adult/CCI, 33.6 ± 1.94%, *p* < 0.01). In addition, there is a substantial increase in the number of neurons positive with 8-oxodG in the middle-aged animals after CCI challenge (Figure 8C, aged/CCI, 1867 ± 188.6 cells per mm^2^ vs. adult/CCI, 1111 ± 173.6 cells per mm^2^, *p* = 0.0025). At the same time, we observed a profound increase in MDA levels (Figure 8D) and hydroxyl free radical (Figure 8E) production after TBI in the middle-aged animals.

To further strengthen the functional significance of thyroid hormones in the modulation of oxidative events that dictate individual TBI-susceptibility, we then applied the *Pearson’s* correlation analyses to understand the functional correlation of individual thyroid function with the levels of oxidative stress. We found both serum levels of T4 (Figure 8F) and T3 (Figure 8G) negatively correlated to the MDA levels and hydroxyl free radical production in the brain. A relatively mild correlation was also observed in the individual thyroid weight to the oxidative events after CCI (Figure 8H). In addition, we measured the mRNA levels of *Sod1, Sod2, Cat* and *Gpx1* by real-time PCR analyses in the middle-aged animals. We found that there is a similar compensatory increase in *Cat* and *Gpx1* after CCI challenge in the adult animals (Figure 8I–L). Remarkably, we found such a protective compensatory increase in the antioxidant genes after CCI challenge disappeared in the middle-aged animals (Figure 8I–L). Again, these results, which recapitulated what we observed in the experiments as surgical thyroidectomy, suggest the functional significance of thyroid hormones in the modulation of compensatory anti-oxidation events and its subsequent TBI susceptibility.

### 3.4. Feasible Therapeutics for Individuals with Thyroid Dysfunction

**Figure 9:**
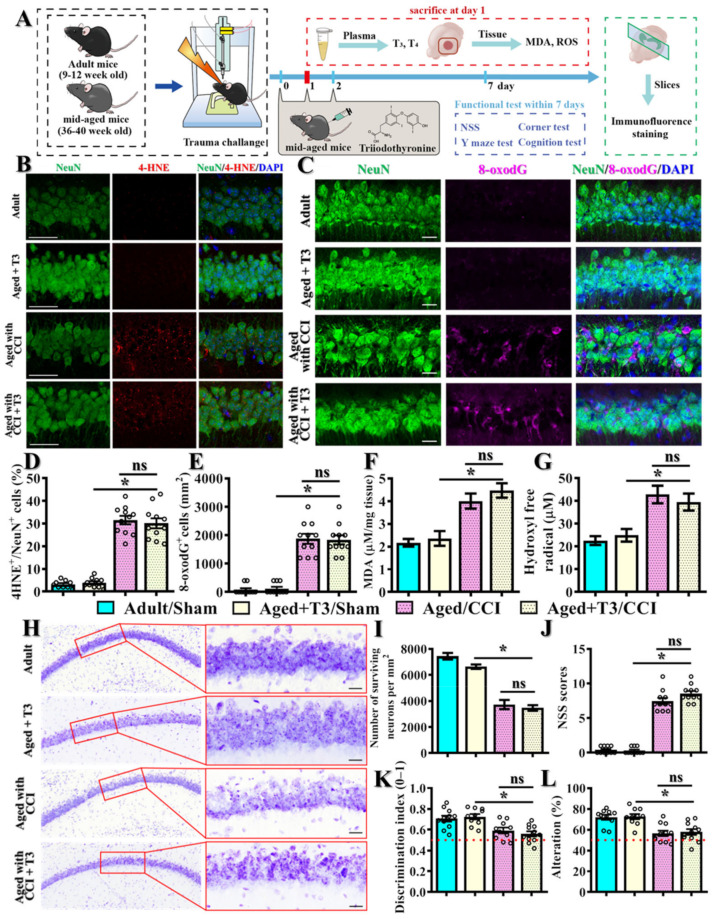
**Acute T3 supplementation has no therapeutic benefits in TBI-susceptible phenotypes.** (**A**) Schematic illustration of the experimental designs with acute T3 supplementation; (**B**) Representative images of 4-HNE-positive (color in red) neurons (NeuN, color in green); (**C**) Representative images of 8-oxodG-positive (color in magenta) neurons (NeuN, color in green). Blue, DAPI signal for the nuclear stain. Scale = 25μm; (**D**) Quantification of the percentage of neurons with 4-HNE-positive puncta; (**E**) Quantification of the regional density of the 8-oxodG-positive neurons. *n* = 15 images from 5 mice in (**D**,**E**); (**F**) Quantification of the levels of malondialdehyde; (**G**) Quantification of the production of hydroxyl free radicals; (**H**–**I**) Representative images of the Nissl stains and quantification analyses showing the surviving neurons in the hippocampal CA1 region. *n* = 15 images from 5 mice; (**J**) Behavioral performance of neurological severity scores. Behavioral performance of cognitive function in the object location recognition task (**K**) and spontaneous alternation Y maze (**L**) from different experimental groups. *n* = 8 to 10 mice. All the data are presented as mean ± SEM and are analyzed by a one-way ANOVA with Bonferroni’s post hoc analysis. * *p* < 0.05; ns, not significant.

Thus far, there are very limited therapeutic options for the treatment of TBI in the clinic, and the situation is even worse for the geriatric TBI patients. Since our data indicates that serum levels of T3 have the best correlation to predict the TBI-susceptible phenotypes after brain trauma, we wondered if the acute supplementation of T3 could ameliorate the TBI-susceptible phenotypes. Experimentally, animals were given 10 mg/kg liothyronine treatment intravenously at 1, 24 and 48 h after CCI challenge and evaluated for their functional recovery from brain trauma (Figure 9A). Through 4-HNE and 8-oxodG staining, we first examined if the acute T3 supplementation could reverse the excessive oxidative stress. To our surprise, T3 supplementation has no effect on the CCI challenge-induced increase in the percentage of neurons with 4-HNE-positive puncta in the middle-aged animals (Figure 9B,D, aged + T3/CCI, 30.1 ± 2.21% vs. aged + vehicle/CCI, 31.55 ± 1.87%). Meanwhile, the T3 supplementation displayed no therapeutic impact on the CCI challenge-induced increase in the number of 8-oxodG-positive neurons in the middle-aged animals after brain trauma (Figure 9C,E, aged + T3/CCI, 1836 ± 159.1 cells per mm^2^ vs. aged + vehicle/CCI, 1873 ± 175.3 cells per mm^2^). At the same time, we observed that there is a significant increase in both MDA (Figure 9F) and hydroxyl free radical (Figure 9G) production after TBI in the middle-aged animals, which indicates that acute T3 supplementation has no significant impact on these markers of oxidative stress.

Meanwhile, Nissl staining was applied to examine the number of surviving neurons at seven days after TBI with the T3 treatment. As demonstrated in Figure 9H,I, the CCI challenge significantly decreased the number of surviving neurons in the hippocampal CA1 region in the middle-aged animals (Figure 9H,I). However, treatment with T3 has no beneficial effect on the CCI challenge-induced decrease in the number of surviving neurons (Figure 9H,I, aged + T3/CCI, 3467 ± 216.5 cells per mm^2^ vs. aged + vehicle/CCI, 3727 ± 351.9 cells per mm^2^).

More importantly, we also evaluated the therapeutic impact of acute T3 supplementation on the functional recovery of motor and cognitive performance one week after CCI challenge (Figure 9A). We found that the CCI challenge resulted in a significant increase in NSS scores in the middle-aged animals with vehicle injection, and T3 treatment has no significant effect on the increased NSS scores after brain trauma (Figure 9J, aged + T3/CCI, 8.545 ± 0.366 vs. aged + vehicle/CCI, 7.455 ± 0.454, *p* = 0.118). Similarly, we found that T3 supplementation has no beneficial impact on the CCI challenge-induced impairment of functional recovery in cognitive performance, which can be reflected in the poorer discrimination in the novel object location test (Figure 9K) and was reduced in the alternation of arm choice in the Y maze task (Figure 9L). These results indicate that acute T3 supplementation might not be able to provide therapeutic benefits in the aged individuals with thyroid dysfunction.

**Figure 10:**
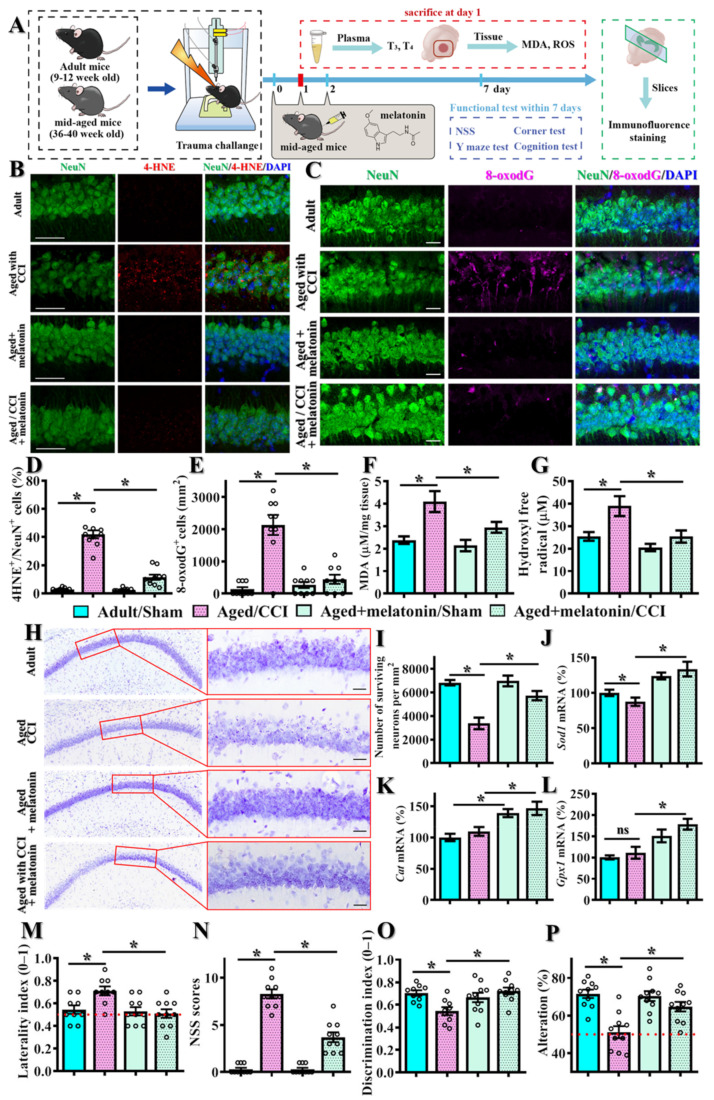
**Amelioration of TBI-susceptible phenotypes by acute melatonin treatment.** (**A**) Schematic illustration of the experimental designs with acute melatonin treatment; (**B**) Representative images of 4-HNE-positive (color in red) neurons (NeuN, color in green); (**C**) Representative images of 8-oxodG-positive (color in magenta) neurons (NeuN, color in green). Blue, DAPI signal for the nuclear stain. Scale = 25 μm; (**D**) Quantification of the percentage of neurons with 4-HNE-positive puncta. (E) Quantification of the regional density of the 8-oxodG-positive neurons. *n* = 15 images from 5 mice in (**D**,**E**); (**F**) Quantification of the levels of malondialdehyde; (**G**) Quantification of the production of hydroxyl free radicals; (**H**–**I**) Representative images of the Nissl stains and quantification analyses showing the surviving neurons in the hippocampal CA1 region. *n* = 15 images from 5 mice; (**J**–**L**) Quantification of the relative expression levels of *Sod1, Cat* and *Gpx1* mRNAs from different experimental groups with or without melatonin treatment. *n* = 5 mice. Behavioral performance of motor function including the corner test (**M**) and neurological severity scores (**N**). Behavioral performance of cognitive function in the object location recognition task (**O**) and spontaneous alternation Y maze (**P**). n = 8 to 10 mice. All the data are presented as mean ± SEM and analyzed by a one-way ANOVA with Bonferroni’s post hoc analysis. * *p* < 0.05; ns, not significant.

As the antioxidant capacity of melatonin is well-known, we then tested if the acute melatonin treatment might provide a neuroprotective impact on the TBI-susceptible phenotypes in the middle-aged individuals. Experimentally, animals were given 10 mg/kg of melatonin treatment intraperitoneally at 1, 24 and 48 h after CCI challenge and evaluated for their functional recovery from brain trauma (Figure 10A). Through 4-HNE and 8-oxodG staining, we first examined if acute melatonin treatment could reverse the excessive oxidative overload in the hippocampus. Interestingly, melatonin treatment significantly reduced the CCI challenge-induced increase in the percentage of neurons with 4-HNE-positive puncta in the middle-aged animals (Figure 10B,D, aged + melatonin/CCI, 11.4 ± 1.875% vs. aged + vehicle/CCI, 41.9 ± 2.78%, *p* = 0.004). At the same time, melatonin treatment also ameliorated the CCI challenge-induced increase in the number of 8-oxodG-positive neurons in the middle-aged animals after brain trauma (Figure 10,E, aged + melatonin/CCI, 440 ± 125.8 cells per mm^2^ vs. aged + vehicle/CCI, 2133 ± 312.7 cells per mm^2^, *p* < 0.001). Meanwhile, we also observed the phenomenon of the CCI challenge that induced an increase in MDA levels (Figure 10F) and hydroxyl free radical production (Figure 10G) in the middle-aged animals were significantly abolished by acute melatonin treatment.

Furthermore, as demonstrated by Nissl staining in Figure 10H,I, the treatment of melatonin significantly abolished the CCI challenge-induced reduction in the number of surviving neurons in hippocampal CA1 from the middle-aged animals (Figure 10H,I, aged + melatonin/CCI, 5733 ± 390.2 cells per mm^2^ vs. aged + vehicle/CCI, 3371 ± 492.7 cells per mm^2^, *p* = 0.0013). In addition, we measured the mRNA levels of the antioxidant genes *Sod1, Sod2, Cat* and *Gpx1* by real-time PCR analyses in the CCI-challenged middle-aged animals after the treatment of melatonin. We found that there is a significant increase in the *Sod1*, Cat and *Gpx1* mRNA expression levels from the melatonin-treated animals after CCI challenge (Figure 10J–L).

More importantly, we also evaluated the therapeutic impact of acute melatonin treatment on the functional recovery of motor and cognitive performance one week after CCI challenge (Figure 10A). We found that the CCI challenge resulted in a significant increase in an asymmetrical turning tendency in the corner test, and acute melatonin treatment significantly normalized the increased asymmetrical turning tendency (Figure 10M, aged + melatonin/CCI, 0.51 ± 0.037 vs. aged+ vehicle/CCI, 0.7111 ± 0.039, *p* = 0.004). In addition, melatonin treatment significantly abolished the CCI challenge-induced increase in NSS scores after brain trauma (Figure 10N, aged + melatonin/CCI, 3.7 ± 0.518 vs. aged + vehicle/CCI, 8.3 ± 0.4726, *p* < 0.001). Similarly, we found melatonin treatment profoundly ameliorated the phenomenon of CCI challenge-induced impairment of functional recovery in cognitive performance, which can be reflected by a better discrimination in the novel object location test (Figure 10O) and an increase in the alternation of arm choice in the Y maze task (Figure 10P) from the middle-aged animals treated with melatonin.

These findings suggest that melatonin could effectively provide anti-oxidative activity and neuroprotection that promotes functional recovery through the induction of antioxidant genes after brain trauma in the aged individuals with thyroid dysfunction.

## 4. Discussion

Traumatic brain injury (TBI) happens when a sudden, external and physical assault damages the brain. As it is one of the most common causes of disability and death in adults, many efforts have been made to understand its pathology. Unfortunately, there is only palliative care available for TBI treatment thus far. The world’s global population is growing and aging, and the elderly population with an age of over 60 years is estimated to reach two billion by 2050. Meanwhile, the number of cases of geriatric TBI is expected to continue to increase in the coming years. There is an urgent need to understand the underlying pathological mechanisms contributing to the impaired functional outcomes and the development of feasible therapeutics for the growing geriatric TBI population.

Instead of using the advanced-age animals, which might exhibit certain pre-existing biological declines that interfere with the pathological investigation, we opted to use middle-aged animals for our current study and found there is an age-dependent difference in TBI susceptibility. Since the individual TBI-susceptibility in the middle-aged animals correlated with their thyroid function, we applied the approach with the surgical removal of thyroid glands. Through the parallel experimental designs by middle-aged animals with surgical thyroidectomy in adult animals, we provide direct evidence showing that there is a thyroid hormone-dependent compensatory regulation of antioxidant events that modulate individual TBI susceptibility. Moreover, we unearth that acute melatonin treatment, but not T3 supplementation, ameliorates oxidative stress, excitotoxic neuronal loss and promotes functional recovery in these aged individuals with thyroid dysfunction.

### 4.1. The Experimental Model with Middle-Aged Animals

Although studies have provided knowledge in the pathological mechanisms of geriatric TBI, most of these studies were conducted through the usage of advanced-age animals [16,17,48]. Thus, the observed impairment in behavioral performance, increase in DNA damage, exaggerated inflammatory responses or oxidative stress overload might be confounded by the existing pre-existing biological function deficits. In our study, the application of the middle-aged animals at the age between 9–12 months old, which display no obvious deficits in pre-TBI behavioral performance and also similar levels in their basal inflammatory and oxidative responses, could provide a more reliable interpretation and solid confirmation of the identified underlying pathology that contributes to the age-dependent difference in TBI susceptibility.

### 4.2. Thyroid Hormone-Dependent Compensatory Protective Events

In addition to the finding of an age-dependent difference in TBI susceptibility, our current study provides a critical finding showing that there is a thyroid hormone-dependent endogenous compensatory regulation of an antioxidant event that modulates the intensity of oxidative stress after CCI. Such an endogenous compensatory mechanism governed by thyroid hormones could ameliorate TBI-induced oxidative overload, aberrant inflammatory responses and eventually contribute to the gradual functional recovery of the brain. Loss of such thyroid hormone-dependent compensatory protective events in the middle-aged animals or animals with surgical thyroidectomy results in the profound TBI-susceptible phenotypes. It has been well documented that thyroid hormones play crucial roles in oxidative regulation, and age-related thyroid dysfunction has been linked to the exaggerative oxidative stress in the pathology of various human diseases. In our current study, we provide further evidence showing how the functional significance of aging-associated thyroid dysfunction could pathologically contribute to the TBI-susceptibility in geriatric individuals.

### 4.3. Melatonin but Not T3 Provides Therapeutic Benefits in Geriatric TBI

Our data indicate that serum levels of T3 exhibit the best correlation to predict the levels of oxidative stress, inflammatory events and functional outcomes. At the beginning, we hypothesized that acute supplementation of T3 could ameliorate the TBI-susceptible phenotypes in the individuals with thyroid dysfunction. To our surprise, there is no significant therapeutic benefit after the acute T3 administration. Few possibilities might contribute to the ineffectiveness of T3 supplementation in the treatment of TBI-related phenotypes in individuals with thyroid dysfunction.

First, both hyperthyroidism and hypothyroidism are associated with oxidative stress in animals and humans. Although extensive studies have indicated that the decrease in thyroid hormones (hypothyroidism) could result in enhanced oxidative stress [49,50,51], paradoxical findings have suggested that the over-production of thyroid hormones also correlated with exaggerated oxidative stress overload [52,53,54]. It is advised that the level of thyroid hormones should be well-tuned to avoid an out-of-control oxidative balance. Second, a recent study showed a significant reduction in the expression levels of thyroid hormone receptors after the surgical removal of thyroid glands [55]. They found that the surgical removal of thyroid glands could result in a significant decrease in various isoforms of thyroid hormone receptors such as *THRα1*, *THRα2* and *THRβ1* mRNA levels after thyroidectomy. Therefore, the loss of therapeutic benefit of acute T3 supplementation could originate from the decrease in the expression of responding thyroid hormone receptors. In addition, it has been well documented that signaling events elicited by thyroid hormones go through their nuclear thyroid hormone receptors. These receptors act as transcription factors that influence target gene expression via binding as heterodimers with retinoid X receptors (RXRs) to the thyroid response elements in the promoter region of T3-responsive genes, including the antioxidant molecules [56]. Therefore, a longer T3 supplementation procedure might be necessary for the transcriptional induction of antioxidant genes to ameliorate TBI-susceptible phenotypes.

On the other hand, plentiful studies have shown that the well-known anti-oxidation compound melatonin could alleviate the pathology of hyperthyroidism-induced oxidative stress and excitotoxic neuronal damage of the brain [57]. Meanwhile, the robust antioxidant capability of melatonin has been reported to originate from its ability to both scavenge oxygen free radicals and also stimulate the production of anti-oxidative enzymes such as superoxide dismutase and glutathione peroxidase. Our results demonstrate that acute melatonin treatment could effectively provide anti-oxidative activity and neuroprotection that promotes functional recovery from brain trauma in the aged individuals with thyroid dysfunction.

### 4.4. Thyroid Function as an Adjuvant Biomarker for Outcome Perdition in Geriatric-TBI

Although the increase in age is one of the important predictors for unfavorable outcomes after brain trauma, a subset of geriatric-TBI individuals recovered well, even after a severe TBI challenge [58,59]. Therefore, the chronological age and initial TBI severity alone might be an inadequate prognostic predictor in geriatric TBI. As world’s elderly population keeps on growing, there is an urgent need to develop evidence-based prognostic indicators which could optimally identify specific elderly individuals who need an earlier therapy or specialized therapeutic strategy. We found that serum levels of thyroid hormones significantly correlated to the functional outcomes, inflammatory indexes and oxidative stress after brain trauma in middle-aged individuals. Therefore, a feasible approach is through the evaluation of serum thyroid hormone levels applied as an adjuvant biomarker for the prognostic prediction of functional outcomes after brain trauma in geriatric-TBI individuals.

## 5. Conclusions

**Figure 11:**
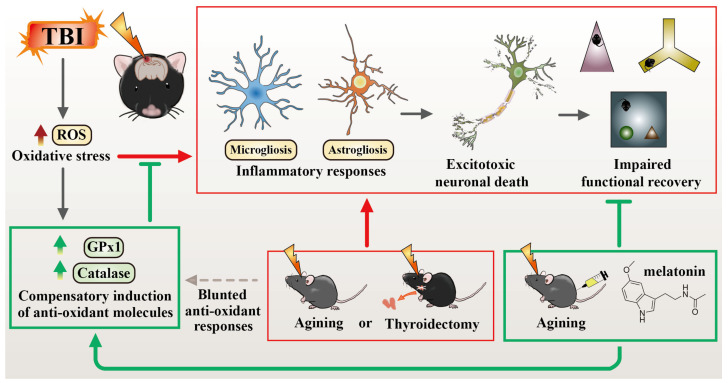
Working model of the major findings in our current study. Numerous pathological events initiated at the acute stage after brain trauma, which contribute to the profound behavioral deficits. Then, the induction of endogenous thyroid hormone-dependent compensatory regulation of anti-oxidant events ameliorates the oxidative stress and inflammatory events that lead to a gradual functional recovery of behavioral performance. Loss of such protective events after surgical removal of the thyroid gland or in aged-associated thyroid dysfunction suppressed the functional recovery of the brain that resulted in TBI-susceptible phenotypes. Events labeled in green color (such as the melatonin treatment) indicate the protective impact on TBI-severity and events labeled in red color (such as aging or thyroidectomy) indicate the adverse impact on TBI-severity.

By using the CCI experiments with middle-aged animals and surgical thyroidectomy, we confirmed that aging-associated thyroid dysfunction pathologically contributes to oxidative stress and worsened functional outcomes following traumatic brain injury (Figure 11). These findings strongly suggest that monitoring thyroid function and acute melatonin treatment could be a feasible therapeutic in the management of geriatric-TBI in clinic.

## Data Availability

All data are available on request from the corresponding author.
